# Identification of Polar Constituents in the Decoction of *Juglans mandshurica* and in the Medicated Egg Prepared with the Decoction by HPLC-Q-TOF MS^2^

**DOI:** 10.3390/molecules22091452

**Published:** 2017-09-01

**Authors:** Tian-Min Wang, Ying Fu, Wen-Jie Yu, Chen Chen, Xue Di, Hui Zhang, Yan-Jun Zhai, Zheng-Yun Chu, Ting-Guo Kang, Hu-Biao Chen

**Affiliations:** 1 School of Pharmacy, Liaoning University of Traditional Chinese Medicine, Dalian 116600, China; 2 School of Chinese Medicine, Hong Kong Baptist University, Kowloon Tong 999077, Hong Kong, China

**Keywords:** *Juglans mandshurica*, constituents, decoction, absorption, medicated eggs, HPLC-Q-TOF-MS^2^

## Abstract

As a folk medicinal plant, *Juglans mandshurica* has been used for the treatment of cancer in China and Korea. Traditionally, *J. mandshurica* is decocted together with chicken eggs. Both the decoction and medicated eggs possess anti-tumor properties. Clarifying the constituents of the decoction and absorbed by the medicated eggs is essential for the investigation of the active principles of *J. mandshurica*. Herein, the medicated eggs were prepared by decocting raw chicken eggs, having unbroken shells, with the decoction of *J. mandshurica*. A systematic investigation of the chemical profile of the *J. mandshurica* decoction and the medicated egg extraction was conducted by HPLC-Q-TOF-MS^2^. In total, 93 peaks, including 45 tannins, 14 naphthalene derivatives, 17 organic acids, 3 diarylheptanoids, 4 lignans, 3 anthraquinones, 1 flavonoid glycoside, 3 amino acids, and 3 nitrogenous compounds, were tentatively identified in the decoction. In the medicated egg extraction, 44 peaks including 11 organic acids, 3 amino acids, 3 nitrogenous compounds, 8 naphthalene derivatives, 3 diarylheptanoids, 15 tannins, and 1 lignan were tentatively identified. The chemical profile presented provided a detailed overview of the polar chemical constituents in *J. mandshurica* and useful information for the research of bioactive compounds of this plant.

## 1. Introduction

The Manchurian walnut, *Juglans mandshurica* Maxim., belongs to the Juglandaceae family and is mainly distributed in northern and northeastern China [1]. It is recorded to have effects of clearing heat, detoxification, astringing lung, and relieving asthma and cough in “Kaibao Bencao” (materia medica edited in the Song dynasty). For its heat clearing and detoxification effects, the decoction of *Juglans mandshurica* roots, bark and immature pericarps has been used for treating cancer [2]. Today, the anti-tumor activity of this plant has been experimentally proven by pharmacological research. As reported, both ethanol and water extracts of *J. mandshurica* roots, bark, branches, leaves, and immature pericarps showed an inhibitory function on the growth of human cancer cell lines (HeLa cervical carcinoma cells and Bel-7402 hepatoma cells) in vitro [3,4] and on implanted murine tumors (H22 hepatoma and S180 solid tumor) in vivo [5,6].

For the research on the active principles of *J. mandshurica*, numerous compounds, including tannins [7,8], flavonoids [9,10], naphthalenes [11], diarylheptanoids [12], organic acids [13], anthracenes [14], triterpenes [15], lignans [16] and phenylpropanoids [17], were isolated from this plant. Some of the compounds isolated have been reported to have growth inhibitory effects on human cancer cell lines (HepG2 hepatoma cells, HL-60 leukemia cells, lung carcinoma cells, SGC-7901 gastric cancer cells, etc.) by the 3-(4,5-dimethylthiazol-2-yl)-2,5-diphenyltetrazolium bromide (MTT) test in vitro [12,13,15,17]. Juglone, which is the main naphthoquinone in *J. mandshurica*, has the most reports of anti-tumor activity. As reported, juglone inhibited growth and induced apoptosis in human HL-60 leukemia cells, HeLa cervical carcinoma cells, and LNCaP prostate cancer cells through different mechanism, including the mitochondria- or reactive oxygen species-dependent pathway and the downregulation of the expression of androgen receptor [18,19]. However, juglone has been also reported to be toxic to human peripheral blood lymphocytes [20], human fibroblasts’ [21] and golden fish [22]. Moreover, it is generally accepted that multiple constituents could be responsible for the biological action in medicinal plants. Therefore, to our knowledge, the active compounds for the anti-tumor activity of *J. mandshurica* have still not been clearly demonstrated.

The discovery of artemisinin from *Artemisia annua* L. (Qing hao) by You-You Tu [23] demonstrates that more attention should be paid to the traditional usage in seeking the active principles of traditional medicine. Most traditional Chinese Medicine was used by decocting. Specially, *J. mandshurica* is decocted together with chicken eggs [24]. As described by Dong and Luo in the journal “Zhongguo Minjian Liaofa”, the medicated eggs should be initially administered and the decoction should be administered when there are no obvious side effects [25]. This traditional usage hints that the decoction and medicated eggs might possess strong and moderate anti-tumor activities, respectively. The anti-tumor activities of the decoction and medicated eggs have been ascertained using the implanted tumor model in mice by our group [26,27].

As is well known, decocting the medicinal plant in boiling water may lead to changes in some constituents of the plant. Our group has revealed that juglone content in *J. mandshurica* obviously decreased with the increase of drying temperature [28]. Juglone has not been detected in the *J. mandshurica* decoction by High performance liquid chromatography (HPLC) analysis [29]. Meanwhile, some constituents like tannins may be absorbed by the eggs decocted with *J. mandshurica* and the compounds absorbed might be effective as anti-tumor compounds. Thus, clarifying the constituents absorbed by the medicated eggs is essential for the investigation of the active principles of *J. mandshurica*. To our knowledge, there are no data on the complete phytochemical profile of the decoction nor the medicated eggs of *J. mandshurica*.

The absence of juglone in the decoction, the anti-tumor activity of the medicated eggs and the ambiguity of the active principles stimulated us to identify the chemical constituents in the decoction of *J. mandshurica* and those absorbed by the medicated eggs decocted with this plant. This multi-constituent identification might provide useful and detailed information on further research of the active principles of *J. mandshurica*. Liquid chromatography coupled to quadrupole time-of-flight mass spectrometry is a powerful tool for rapid characterization of multiple compounds from traditional Chinese medicine [30]. In the present paper, high performance liquid chromatography with diode array detector coupled with quadrupole time-of-flight mass/mass spectrometry through electrospray ionization interface (HPLC-DAD-Q-TOF-MS^2^) was applied for multi-constituent identification.

## 2. Results and Discussion

### 2.1. Optimization of the Sample Preparation Procedure and the HPLC-Q-TOF-MS Conditions

Referring to the traditional usage of *J. mandshurica*, the decoction was prepared by decocting branches of *J. mandshurica* in water. The medicated eggs were obtained by decocting them with the *J. mandshurica* decoction. After decocting, the color of the medicated eggs were dark brown due to the absorption of the constituents in the decoction of *J. mandshurica*. For thorough extraction of the constituents absorbed by the medicated eggs, the medicated eggs were lyophilized immediately after preparation to remove water in them and to obtain a fine powder, which increased the contact area with the extraction solvent and, therefore, improved the extraction efficiency. As previously reported by our group [31], the conditions for the extraction of the constituents absorbed by the medicated eggs, including the extraction method, extraction solvent, its volume and the extraction time, were optimized. Herein, the extraction conditions utilized were consistent with those optimized in the literature [31].

The decoction and the medicated and blank egg solutions were all subjected to HPLC-Q-TOF-MS^2^ analysis. HPLC-Q-TOF-MS conditions were optimized to obtain good separation and an abundant response of multiple compounds in the decoction of *J. mandshurica*. Mobile phases consisting of acetonitrile and water both with 0.2% formic acid (*v*/*v*) were applied to obtain sharp peak shape for most phenolic compounds and a relatively stable baseline in the UV detector. The chromatographic separation efficiency of three different reversed-phase C18 columns, namely Agilent Zorbax SB-C18 (4.6 × 250 mm, 5 μm), Waters Spherisord ODS1 (4.6 × 250 mm, 5 μm) and Waters μBondapak C18 (3.9 × 300 mm, 10 μm), was evaluated for HPLC. The Agilent Zorbax SB-C18 column eluted with a gradient of acetonitrile-water with 0.2% formic acid (*v*/*v*) gave a good chromatographic separation for most constituents in the *J. mandshurica* decoction. MS spectra were conducted under negative-ion mode according to the literature [32] on the MS behavior of most of the phenolic compounds. To get almost all information on the chemical profile of the *J. mandshurica* decoction, non-targeted auto MS/MS was employed. The collision energy in MS/MS was optimized, and an energy of 20 eV was finally applied.

### 2.2. Identification of Compounds in Juglans Mandshurica Decoction

**Figure 1:**
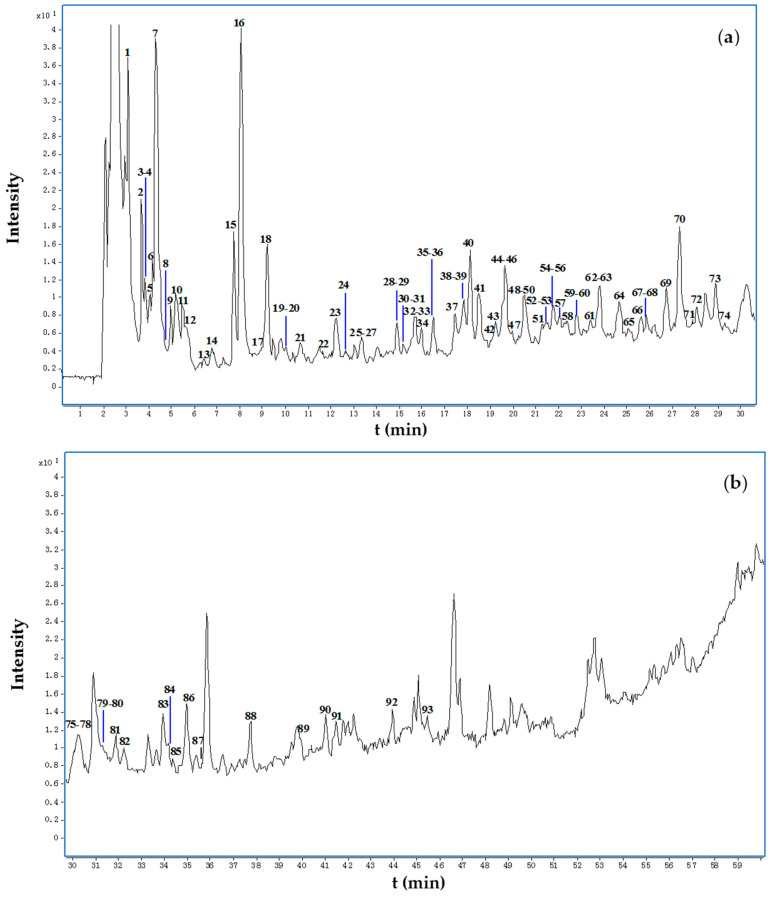
Total ion chromatograms (TIC) in negative-ion mode (from HPLC-ESI-Q-TOF-MS) of the *Juglans mandshurica* decoction (**a**) within 0–30 min and (**b**) 30–60 min. The peak numbering in each TIC relates to the numbered compounds listed in Table 1.

The total ion chromatograms (TIC) of the *J. mandshurica* decoction obtained by HPLC-ESI-Q-TOF-MS in negative-ion mode are shown in Figure 1. Multiple constituents were characterized by accurate mass analysis of the precursor and product ions and comparing their fragmentation patterns with those of reference compounds or those reported in the literature. As shown in Table 1, 93 peaks, in which configuration isomers were included, were tentatively identified in the *J. mandshurica* decoction. Those compounds with stereo centers which configuration can’t be determined only by MS data was tentatively identified as the one that universally exist as natural products. For example, hexose present in tannins was characterized as glucose and 2,3,4-trihydroxybutanoic acid was characterized as threonic acid. The compounds identified consisted of tannins, naphthalene derivatives, organic acids, flavonoids, diarylheptanoids, lignans, and anthraquinones. The result and the fragmentation pattern of those compounds identified was consistent with our previous report on the chemical profile of ethanol extract of *J. mandshurica* [33].

Tannins were found to be the main constituents in the decoction and 45 tannins were characterized. The fragmentation pattern of these tannins was characterized by continuous loss of acyl and/or organic acid groups and presence of typical product ions of the corresponding organic acid (Figure S1a) [32,33]. The organic acid and the polyol carbohydrate core in the structure of the tannins identified mainly consisted of gallic acid and glucose, respectively. Additionally, methyl gallic acid, syringic acid, vanillic acid, and phenol derivatives (as the polyol carbohydrate core) were also found in the structure of the identified tannins. These organic acids [34,35] and phenol derivatives [11,36,37] or compounds with these groups [38] have been reported to be found in *J. mandshurica*. More mono- and di-*O*-galloyl glucoses other than tetra-*O*-galloyl glucoses, which have been reported to commonly exist in *J. mandshurica*, were found in the decoction of *J. mandshurica*. This observation suggested that decocting in boiling water resulted in the hydrolysis of tannins with more galloyl groups [39].

Both naphthoquinones, for example juglone [18], and naphthalene derivatives [11,35,40] were reported to exist in *J. mandshurica*. However, no naphthoquinone, but naphthalene derivatives, including 11 hydroxyl tetralone derivatives and three hydroxyl naphthalene derivatives were found in the *J. mandshurica* decoction. Based on this evidence, the anti-tumor effects of the *J. mandshurica* decoction might not be due to juglone alone. The absence of naphthoquinones in the decoction might be caused by volatilization during decocting [41]. Of the naphthalene derivatives identified, nine were glycosides with glycosyl groups or galloyl/syrigoyl-substituted glycosyl moieties. According to MS^2^ analysis, fragmentation of these naphthalene derivatives involved the loss of H_2_O and CO_2_ from deprotonated aglycon ions (Figure S1b) [33,37].

Besides tannins and naphthalenes, organic acids were also the main compounds existing in *J. mandshurica*. Typical fragmentation of these organic acids involved the loss of CO_2_ (Figure S1c), which was consistent with the results reported in the literature. Herein, 17 organic acids were identified by comparing their MS data with those reported in the literature [42,43,44]. Organic acids that are present in the structure of tannins, such as gallic acid, syringic acid, and ellagic acid, were included. Additionally, compounds with groups of coumaric acid, caffeic acid, ferulic acid and quinic acid, which have been reported to be found in *J. mandshurica* [45,46], were also included in the organic acids identified. However, some compounds with threonic acid (Peak 48, 57, 59, 70, 74 and 82), which has not been found in *J. mandshurica*, were tentatively identified in this plant for the first time.

Flavonoids [9], diarylheptanoids [47], lignans [48] and anthraquinones [14,49], which were reportedly isolated from *J. mandshurica*, were also detected in the decoction. One flavonoid, namely quercetin-3-*O*-α-l-rhamnoside, was identified by its identical retention time and fragment pattern with the reference compound. One lignan, together with its pentoside, was identified by comparing its MS data with that reported in the literature [50]. In addition, three diarylheptanoids (Peaks 86, 89, and 90), two lignans (Peaks 38 and 92), and three anthraquinones (Peaks 87, 91, and 93) were tentatively identified in the decoction due to the lack of reference compounds and mass data in the literature. Accurate mass analysis and retrieval in SciFinder for possible compounds, which was consistent with the MS^2^ data, were employed in those tentative identifications. The report of isolation of peaks 38 [51], 86 [2], 87 [14], 89 [52], and 90 [53] form Juglans genus made the tentative identification of these compounds more reasonable. The MS^2^ data and proposed fragmentation pattern of those eight compounds tentatively identified were supplied as supplementary materials (Figure S2).

Six nitrogenous compounds, namely uric acid (Peak 4), uridine (Peak 9), tyrosine (Peak 11), phenylalanine (Peak 19), pantothenic acid (Peak 22), and tryptophan (Peak 33), were identified in the decoction. These compounds, with some of which are mainly found in the metabolites of animals, have been proven to exist in plants [44]. The fragmentation patterns and even the retention order in the column of these compounds were consistent with those in the literature [44].

### 2.3. Identification of Compounds in Medicated Eggs Decocted with the Juglans Mandshurica Decoction

For the identification of constituents absorbed by the medicated eggs, the TIC of medicated and blank egg solutions were fully compared and peaks that were present in both the medicated egg solution and the decoction, but absent in the blank egg solution, were ascertained to be constituents absorbed from the decoction. Those peaks only present in the medicated egg solution, but absent in both the blank egg solution and the decoction, which might have been caused by the chemical changes of the eggs after the interaction with *J. mandshurica*, were not taken into account in this paper.

**Figure 2:**
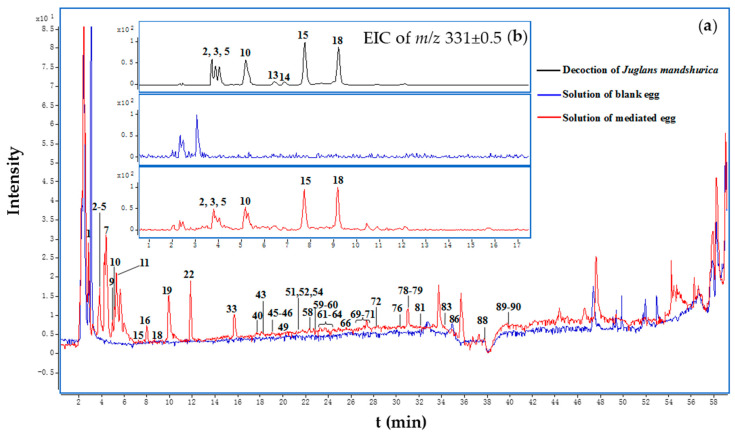
(**a**) Total ion chromatograms (TIC) in negative-ion mode (from HPLC-ESI-Q-TOF-MS) of medicated and blank egg solutions and (**b**) extracted ion chromatograms (EIC) of the *Juglans mandshurica* decoction and blank and medicated egg solutions at *m*/*z* 331 ± 0.5 in MS. The peak numbering in TIC and EIC relates to the numbered compounds listed in Table 1.

When decocted with eggs, the constituents in the decoction were selectively absorbed by the eggs. The constituents absorbed by the medicated eggs were extracted with 70% acetone (*v*/*v*) by reflux. The extraction was subjected to HPLC-Q-TOF-MS^2^ analysis, and the TIC of medicated and blank egg solutions are shown in Figure 2a. By comparing the TIC, extracted ion chromatograms (EIC, Figure 2b, Figures S3a and S4) and MS/MS data (Figure S3b) of the medicated egg solutions with those of the blank egg solutions and decoction, 44 peaks were identified as absorbed constituents. These absorbed peaks included 11 organic acids, 3 amino acids, 3 nitrogenous compounds, 8 naphthalene derivatives, 3 diarylheptanoids, 15 tannins and 1 lignan. Except for flavonoids and anthraquinones, many kinds of compounds detected in the decoction were absorbed. Most compounds were absorbed in a concentration dependent manner. An obvious selectivity was observed for tannins. Only tannins with less than two galloyl groups (or equivalent groups) were detected in the medicated egg solutions. For example, several compounds of mono-*O*-galloyl-glucose were found (Figure 2b), but no di-*O*-galloyl-glucose was detected (Figure S4) in the medicated egg solutions. According to the property that tannins co-predicate with protein, the absence of di-*O*-galloyl-glucose might have been partially due to its binding with protein. At the same time, these big molecular tannins might not be absorbed by the eggs. For confirmation, further investigation of the reference tannins will be required.

The absorption ratio of constituents absorbed by the medicated eggs was determined by semi-quantitative analysis. The concentration of the decoction for analysis and for the preparation of medicated eggs remained constant and was 0.1 g/mL for the crude drug. The concentration of the medicated egg solutions for analysis was 0.22 g/mL for the crude drug. According to the HPLC-DAD analysis, the reproducibility of the retention time and peak area for the decoction (obtained from six decoction samples) and the medicated egg solutions (obtained from three egg samples) was found to be relatively consistent, with an RSD of less than 7%. The reproducibility of the peak area was important for semi-quantitative analysis of the absorption ratio of the constituents absorbed by the medicated eggs. The absorption ratio was calculated using the following equation:

$$\mathrm{Absorption}\;\mathrm{ratio}\;(\%)=\frac{\mathrm{Peak}\;\mathrm{area}\;\mathrm{in}\;\mathrm{medicated}\;\mathrm{egg}\;\mathrm{solution}\times 0.1}{\mathrm{Peak}\;\mathrm{area}\;\mathrm{in}\;\mathrm{decoction}\times 0.22}\times 100\%$$

As obtained by semi-quantitative analysis, the absorption ratio of most absorbed compounds was within the range of 1.0–9.0% (Table 1). The absorption ratios of three amino acid, uric acids, uridine, pantothenic acid and citric acid were >15%, with the biggest being 1706.8% (tyrosine). Most of these compounds also existed in eggs in different forms; for example, amino acids were combined as protein [54]. During decocting, the interaction of the eggs and the *J. mandshurica* decoction might have led to the chemical changes in the eggs, and therefore those aforementioned compounds were produced and extracted from the medicated eggs, which resulted in a large absorption ratio.

## 3. Materials and Methods

### 3.1. Chemicals

Acetone (AR grade) used for extraction was purchased from Tianjin Kemiou Chemical Reagent Co., Ltd. (Tianjin, China) HPLC grade acetonitrile, methanol, and formic acid utilized in HPLC-MS analysis were purchased from Honeywell (Morris, NJ, USA). Water used for the HPLC mobile phase and extraction solvent was purchased from Hangzhou Wahaha Group Co., Ltd. (Hangzhou, China).

### 3.2. Plant Materials

Branches of *Juglans mandshurica* Maxim. were collected from “Laobai Shan” nearby Lianshan village of Pulandian county, Liaoning Province, northeast China, in May 2013. The identity of the plant species was confirmed by Prof. Yan-Jun Zhai. The fresh samples were sliced into approximately 0.5 cm pieces and dried at room temperature (20–23 °C) in the shade. Dry raw materials were stored at room temperature in sealed plastic bags before analysis. A voucher specimen (Zhi130503) was deposited in the Herbarium of Liaoning University of Traditional Chinese Medicine.

### 3.3. Reference Compounds and the Preparation of Reference Solutions

Reference compounds of gallic acid, protocatechuic acid, chlorogenic acid, caffeic acid, syringic acid, *p*-coumaric acid, ellagic acid, sinapic acid and juglone with a purity >98% (determined by HPLC-UV analysis) were purchased from Acros organics. 1,2,6-tri-*O*-galloyl-β-d-glucose, 1,2,3,6-tetra-*O*-galloyl-β-d-glucose and 1,4,8-trihydroxy-naphthalene 1-*O*-β-d-glucoside were isolated from ethanol extract of *J. mandshurica* by semi-preparative HPLC (YMC-Pack C18 column, 250 mm ×10 mm, 5 μm) using a MeOH-H_2_O containing 0.5% (*v*/*v*) formic acid (35:65) for elution. Kaempferol, quercetin, acacetin, rutin, apigenin, luteolin, kaempferol 3-*O*-β-d-glucoside and quercetin 3-*O*-β-d-glucoside were isolated from *Saussurea stella* Maxim. by Prof. Shao-Qing Cai and Dr. Tian-Min Wang [55]. The chemical structures of the isolated compounds were ascertained by comparing their NMR and MS spectral data with those reported in the literature [38,55,56]. The purity of each compound was more than 95% as determined by HPLC-DAD analysis. These 20 reference compounds were divided into two groups according to their retention time. Each compound was dissolved in 50% (*v*/*v*) methanol (1 mg/mL) to get the stock solution and stored at −4 °C. Prior to HPLC-Q-TOF-MS analysis, an appropriate volume of the stock solutions in each group was mixed to get mixed standard solution A and B as the working solutions. The concentration of each compound in the working solutions ranged from 10 to 116 μg/mL (Table S1).

### 3.4. Preparation of the Decoction of Juglans Mandshurica

Dry branches of *J. mandshurica* were ground into a powder, weighed (100 g), and decocted in water at a 1:10 ratio (*w*/*w*) of plant material to solvent. During decocting, the appropriate amount of water was occasionally added to maintain a constant quantity of water. After decocting for 2 h, the decoction together with the plant material decocted were weighed, and water was added to accurately recover the weight loss. The decoction was separated by centrifugation at 1500× *g* for 5 min. After filtration through a 0.45 μm syringe filter (Agilent Technologies, Palo Alto, CA, USA), the decoction was subjected to HPLC-Q-TOF-MS^2^ analysis.

### 3.5. Preparation of Blank and Medicated Eggs and Blank and Medicated Egg Solutions

Chicken eggs were purchased from Tesco supermarket. For the preparation of medicated eggs, one of the raw eggs with an unbroken shell was weighed and decocted in *J. mandshurica* decoction (1:10, *w*/*v*) prepared by the aforementioned method. For the preparation of blank eggs, one of the raw eggs with an unbroken shell was weighed and decocted in water (1:10, *w*/*v*). The decocting procedure of these two eggs was the same as that of the powder of *J. mandshurica* branches. After the addition of water to recover the weight loss, these two eggs were pulled out and their shells discarded. The unshelled eggs were separately rinsed and blended with water. The mixture of ground egg and water was frozen and lyophilized by an ALPAI1-4/LSC freeze drier purchased from Marin Christ Corporation (Osterode am Harz, Germany) at −50 °C under 6 Pa to get a fine powder of blank eggs (16.7%, *w*/*w*) and medicated eggs (18.3%, *w*/*w*).

Powder (0.5 g) of the blank and medicated eggs was separately extracted with 25 mL of 50% acetone (*v*/*v*) under reflux for 2 h. The extraction was filtrated, and 10 mL of the filtration were dried at room temperature using a nitrogen evaporator. The residue was redissolved in 5 mL of 10% methanol (*v*/*v*). Prior to injection, the solution was filtrated through 0.45 μm syringe filters (Agilent Technologies, Palo Alto, CA, USA).

### 3.6. HPLC-Q-TOF-MS^2^ System and Conditions

LC-MS analysis was conducted on an Agilent 1290 HPLC-DAD system connected to an Agilent 6540 Q-TOF mass spectrometry (Agilent Technologies, Palo Alto, CA, USA) by an electrospray ionization (ESI) interface. Liquid chromatographic separation was performed on an Agilent Zobax SB C18 column (5 μm, 4.6 × 250 mm) maintained at 30 °C and eluted with gradient water (A) and acetonitrile (B), both with 0.2% (*v*/*v*) formic acid at a flow rate of 1.0 mL/min. The following gradient elution was applied: 3% B between 0 and 3 min, from 3 to 25% B between 3 and 35 min, from 25 to 60% B between 35 and 55 min, from 60 to 95% B between 55 and 60 min, from 95 to 100% B between 60 and 61 min. The injection volume was 10 μL for reference solutions and 20 μL for other analytes. The effluent from HPLC-DAD was drained to the MS system with a split ratio of 4:1.

Mass spectra were operated in negative-ion mode. The source ionization conditions were as follows: drying and sheath gas temperature, 350 °C; drying and sheath gas flow rate, 8.0 L/min; nebulizer, 35 psi; capillary voltage, 3500 V; fragmentor voltage, 75 V; skimmer voltage, 65 V. Auto MS/MS targeted three maximum precursor ions per acquisition cycle using collision energy of 20 eV. Data were acquired over a range of *m*/*z* 100–2000 for MS and *m*/*z* 50–2000 for MS/MS with an acquisition rate of 1 spectra/second. Spectra were processed using Agilent MassHunter Qualitative Analysis.

## 4. Conclusions

In summary, a systematic investigation of the chemical profile of the *J. mandshurica* decoction and the medicated egg extraction was conducted by HPLC-Q-TOF-MS^2^. Consequently, 93 peaks were identified in the decoction, while 44 peaks were identified in the solvent extraction of medicated eggs. The chemical profile provided a detailed overview of the chemical constituents in *J. mandshurica*. The detailed mass data presented in this paper provide useful information on the rapid identification of natural products from the title plant and even other plants. The decoction and medicated eggs of *J. mandshurica* were reported to possess anti-tumor activity; thus, the identification of the chemical compounds in the decoction and the medicated egg extraction adds significantly to our further research on the bioactive constituents of *J. mandshurica*.

## Figures and Tables

**Table 1:** Constituents tentatively identified in *Juglans mandshurica* decoction and those absorbed by medicated eggs using HPLC-Q-TOF-MS^2^.

Peak	t_R_ (min)	*m*/*z* ^1^, [M − H]^−^		Formula	Major Fragments (Negative-Ion Mode) ^2^	Identification ^3^	Absorbed by Medicated Eggs		
		Measured	Mass Error (ppm)				Peak Area in Medicated Eggs Solution (×10^3^)	Peak Area in Decocotion (×10^3^)	Absorption Ratio (%) ^4^
1	3.04	133.0143	4.51	C_4_H_6_O_5_	115.0025 (14), 89.0258 (9), 71.0139 (100)	Malic acid	794	18,028	2.0
2	3.71	331.0677	3.62	C_13_H_16_O_10_	211.0278 (18), 169.0135 (81), 125.0238 (100), 124.0154 (60), 107.0132 (34), 89.0247 (18)	Mono-*O*-galloyl-glucose	41	1526	1.2
3	3.84	331.0672	2.11	C_13_H_16_O_10_	211.0225 (41), 169.0132 (78), 125.0233 (60), 124.0165 (100), 107.0138 (56), 89.0259 (7)	Mono-*O*-galloyl-glucose	20	870	1.0
4	3.86	167.0204	−0.60	C_5_H_4_N_4_O_3_	124.0144 (100), 96.0200 (49), 69.0098 (30)	Uric acid	1163	331	159.7
5	4.05	331.0668	0.91	C_13_H_16_O_10_	211.0216 (10), 169.0112 (100), 125.0234 (89), 124.0160 (91), 107.0120 (36)	Mono-*O*-galloyl-glucose	28	1094	1.2
6	4.18	481.0626	1.66	C_20_H_18_O_14_	300.9978 (100), 275.0194 (38), 257.0072 (14)	Mono-*O*-HHDP-glucose ^5^			
7	4.30	191.0199	3.66	C_6_H_8_O_7_	111.0088 (72), 87.0087 (100), 85.0291 (49), 67.0184 (29), 57.0346 (33)	Citric acid	9267	25,918	16.3
8	4.68	481.0626	1.66	C_20_H_18_O_14_	300.9948 (100), 275.0205 (34), 257.0117 (13)	Mono-*O*-HHDP-glucose ^5^			
9	4.97	243.0617	0.00	C_9_H_12_N_2_O_6_	152.0329 (16), 110.0244 (100)	Uridine	282	738	17.4
10	5.23	331.0676	3.32	C_13_H_16_O_10_	271.0466 (8), 211.0237 (26), 169.0122 (56), 125.0230 (67), 124.0155 (53), 107.0112 (20), 59.0136 (100)	Mono-*O*-galloyl-glucose	161	3721	2.0
11	5.44	180.0668	3.89	C_9_H_11_N_1_O_3_	163.0387 (46), 119.0496 (100), 93.0347 (39)	Tyrosine	3755	100	1706.8
12	5.60	481.0626	1.66	C_20_H_18_O_14_	300.9983 (100), 275.0184 (57), 257.0088 (14)	Mono-*O*-HHDP-glucose ^5^			
13	6.49	331.0678	3.93	C_13_H_16_O_10_	271.0520 (13), 211.0289 (33), 169.0171 (94), 125.0266 (18), 124.0154 (36), 107.0208 (19), 59.0153 (100)	Mono-*O*-galloyl-glucose			
14	6.91	331.0664	−0.30	C_13_H_16_O_10_	169.0145 (79), 124.0203 (36), 107.0140 (9)	Mono-*O*-galloyl-glucose			
15	7.75	331.0676	3.32	C_13_H_16_O_10_	271.0431 (39), 211.0238 (45), 169.0129 (32), 125.0225 (40), 124.0160 (100), 107.0137 (16)	Mono-*O*-galloyl-glucose	214	5022	1.9
16 ^6^	8.04	169.0150	7.69	C_7_H_6_O_5_	125.0238 (100), 124.0162 (11), 107.0134 (5), 97.0285 (10), 79.0186 (21)	Gallic acid	1862	23,485	3.6
17	9.13	483.0779	0.83	C_20_H_20_O_14_	331.0655 (17), 313.0516 (14), 271.0472 (6), 169.0139 (100), 125.0237 (85), 107.0118 (13)	Di-*O*-galloyl-glucose			
18	9.22	331.0687	6.65	C_13_H_16_O_10_	271.0464 (37), 211.0237 (54), 169.0137 (47), 125.0233 (57), 124.0162 (100), 107.0142 (25)	Mono-*O*-galloyl-glucose	185	3901	2.2
19	10.06	164.0714	0.00	C_9_H_11_N_1_O_2_	147.0445 (28), 103.0551 (100), 91.0565 (14), 72.0093 (55)	Phenylalanine	2962	118	1141.0
20	10.06	483.0775	0.00	C_20_H_20_O_14_	331.0746 (8), 313.0608 (6), 271.0466 (5), 169.0142 (94), 125.0225 (100)	Di-*O*-galloyl-glucose			
21	10.64	483.0777	0.41	C_20_H_20_O_14_	331.0697 (8), 211.0259 (6), 169.0129 (74), 125.0248 (100)	Di-*O*-galloyl-glucose			
22	11.82	218.1030	0.92	C_9_H_17_N_1_O_5_	146.0814 (52), 88.0405 (100)	Pantothenic acid	626	327	87.0
23	12.24	483.0789	2.90	C_20_H_20_O_14_	331.0710 (14), 313.0564 (20), 271.0411 (12), 211.0240 (19), 169.0134 (99), 125.0249 (100), 124.0166 (9)	Di-*O*-galloyl-glucose			
24	12.62	329.0881	2.43	C_14_H_18_O_9_	167.0304 (55), 152.0190 (66), 123.0436 (64), 108.0208 (100)	Mono-*O*-vanilloyl-glucose			
25	13.29	483.0791	3.31	C_20_H_20_O_14_	211.0283 (29), 169.0138 (100), 125.0223 (43), 124.0134 (14)	Di-*O*-galloyl-glucose			
26	13.33	345.0829	2.03	C_14_H_18_O_10_	183.0261 (23), 138.0315 (100)	Methylgalloyl-*O*-glucose			
27	13.37	359.0983	1.39	C_15_H_20_O_10_	344.0747 (75), 197.0379 (33), 166.9989 (20), 137.0251 (100)	Mono-*O*-syringoyl-glucose			
28	14.84	345.0812	−2.90	C_14_H_18_O_10_	138.0304 (100)	Methylgalloyl-*O*-glucose			
29	14.93	483.0796	4.35	C_20_H_20_O_14_	271.0465 (41), 211.0243 (69), 169.0135 (100), 125.0236 (47), 124.0157 (69)	Di-*O*-galloyl-glucose			
30	15.18	359.0979	0.28	C_15_H_20_O_10_	344.0754 (48), 197.0475 (79), 182.0225 (26), 166.9989 (13), 152.0455 (50), 137.0251 (84), 123.0089 (100)	Mono-*O*-syringoyl-glucose			
31	15.31	353.0864	−2.55	C_16_H_18_O_9_	191.0571 (100), 179.0288 (40), 135.0440 (78), 117.0370 (25)	Caffeoyl quinic acid			
32	15.68	633.0759	4.90	C_27_H_22_O_18_	481.0643 (8), 463.0506 (7), 300.9996 (100), 275.0192 (27), 169.0113 (7)	HHDP-galloyl-*O*-glucose ^5^			
33	15.81	203.0819	−0.98	C_11_H_12_N_2_O_2_	142.0642 (29), 116.0500 (100), 74.0249 (24)	Tryptophan	1443	178	368.5
34	15.98	483.0793	3.73	C_20_H_20_O_14_	423.0526 (34), 331.0733 (5), 313.0620 (11), 271.0455 (32), 211.0238 (52), 169.0135 (100), 125.0234 (86)	Di-*O*-galloyl-glucose			
35	16.44	329.0875	0.61	C_14_H_18_O_9_	269.0721 (19), 209.0434 (35), 167.0343 (41), 152.0111 (15), 123.0435 (24), 59.0136 (100)	Mono-*O*-vanilloyl-glucose			
36	16.48	483.0787	2.48	C_20_H_20_O_14_	313.0563 (6), 271.0452 (17), 211.0233 (41), 169.0134 (100), 125.0241 (62)	Di-*O*-galloyl-glucose			
37	17.41	483.0799	4.97	C_20_H_20_O_14_	423.0596 (23), 331.0711 (6), 313.0509 (5), 271.0449 (26), 211.0246 (21), 169.0128 (100), 125.0246 (55)	Di-*O*-galloyl-glucose			
38 ^7^	17.83	357.1183	−0.84	C_16_H_22_O_9_	177.0541 (8), 133.0659 (100)	Juglanoside H			
39	17.83	483.0787	2.48	C_20_H_20_O_14_	331.0651 (6), 313.0562 (14), 271.0452 (24), 211.0245 (29), 169.0142 (100), 125.0240 (68), 107.0138 (12)	Di-*O*-galloyl-glucose			
40	18.12	359.0990	3.34	C_15_H_20_O_10_	299.0759 (8), 239.0560 (25), 197.0448 (54), 182.0209 (12), 166.9973 (5), 152.0465 (15), 137.0239 (24), 59.0135 (100)	Mono-*O*-syringoyl-glucose	105	3282	1.5
41	18.50	483.0798	4.76	C_20_H_20_O_14_	313.0615 (14), 271.0435 (51), 211.0239 (77), 169.0127 (100), 125.0237 (71), 107.0144 (15)	Di-*O*-galloyl-glucose			
42	19.13	511.1098	1.96	C_22_H_24_O_14_	467.1314 (15), 327.0605 (14), 313.0528 (40), 197.0495 (10), 182.0226 (14), 169.0138 (67), 125.0228 (100), 124.0172 (31), 107.0121 (18)	Syringoyl-galloyl-*O*-glucose			
43	19.25	483.1141	0.41	C_21_H_24_O_13_	327.0770 (8), 313.0573 (6), 297.0666 (11), 169.0473 (6), 169.0129 (78), 154.0254 (14), 139.0041 (8), 125.0228 (70), 124.0160 (100), 107.0134 (13)	Hydroxy-dimethoxyphenol galloyl-glucoside	51	785	3.0
44	19.51	483.0767	−1.66	C_20_H_20_O_14_	331.0609 (9), 313.0567 (58), 271.0450 (39), 211.0244 (25), 169.0130 (100), 125.0250 (47)	Di-*O*-galloyl-glucose			
45 ^6^	19.59	353.0880	1.98	C_16_H_18_O_9_	191.0547 (100), 135.0437 (8), 93.0353 (22)	Chlorogenic acid	26	798	1.5
46	19.67	359.0994	4.46	C_15_H_20_O_10_	197.0430 (29), 182.0203 (8), 166.9953 (5), 152.0455 (24), 137.0237 (42), 59.0138 (100)	Mono-*O*-syringoyl-glucose	72	1953	1.7
47	19.97	635.0859	−3.94	C_27_H_24_O_18_	483.0814 (10), 465.0776 (10), 313.0566 (10), 295.0513 (8), 271.0492 (6), 169.0134 (100), 125.0257 (11)	Tri-*O*-galloyl-glucose			
48	20.47	281.0669	2.85	C_13_H_14_O_7_	163.0402 (100), 119.0492 (100), 75.0084 (33)	Coumaroyl threonic acid			
49	20.51	533.1494 ^8^	−2.25	C_21_H_28_O_13_	193.04490 (100), 175.0377 (42)	Trihydroxytetralone pentosyl-hexoside	13	259	2.4
50	20.60	453.1051	3.97	C_20_H_22_O_12_	313.0523 (27), 169.0121 (23), 139.0410 (14), 125.0229 (27), 124.0160 (100), 97.0300 (8)	hydroxy-methoxyphenol galloylglucoside			
51	21.27	291.0139	−0.69	C_13_H_8_O_8_	247.0245 (100), 219.0288 (13), 191.0341 (37), 173.0250 (14), 145.0280 (21)	Brevifolin carboxylic acid	59	1330	2.0
52	21.48	533.1516 ^8^	1.88	C_21_H_28_O_13_^−^	193.0505 (100), 175.0409 (24)	Trihydroxytetralone pentosyl-hexoside	16	203	3.4
53	21.56	339.1076	−1.18	C_16_H_20_O_8_	159.0430 (93), 115.0552 (100)	Dihydroxytetralone hexoside			
54	21.73	533.1520 ^8^	2.63	C_21_H_28_O_13_	193.0502 (100), 175.0386 (30)	Trihydroxytetralone pentosyl-hexoside	48	623	3.5
55	21.81	401.1084 ^8^	0.00	C_16_H_20_O_9_	193.0506 (100), 175.0390 (42)	Trihydroxytetralone hexoside			
56	21.90	635.0888	0.63	C_27_H_24_O_18_	483.0850 (52), 465.0636 (45), 169.0140 (100), 125.0244 (11)	Tri-*O*-galloyl-glucose			
57	22.11	281.0661	0.00	C_13_H_14_O_7_	163.0390 (23), 119.0501 (100), 117.0208 (56), 75.0103 (42)	Coumaroyl threonic acid			
58	22.40	481.0998	3.33	C_21_H_22_O_13_	313.0551 (53), 169.0143 (100), 167.0323 (38), 152.0104 (45), 125.0234 (63), 124.0149 (18)	Vanilloyl-*O*-galloyl-glucose	45	710	2.9
59	22.86	297.0614	1.35	C_13_H_14_O_8_	179.0301 (26), 161.0284 (16), 135.0284 (96), 107.0497 (23), 75.0077 (100)	Caffeoyl threonic acid	30	564	2.4
60 ^6^	22.86	197.0444	−3.04	C_9_H_10_O_5_	182.0228 (14), 166.9941 (100), 123.0085 (65), 95.0135 (34), 61.9890 (53)	Syringic acid	86	479	8.2
61	23.41	467.0812	−3.00	C_20_H_20_O_13_	423.0881 (62), 315.0710 (23), 313.0557 (33), 169.0144 (69), 153.0205 (27), 152.0107 (97), 125.0231 (78), 109.0281 (33), 108.0209 (100)	Dihydroxybenzoic acid galloyl-glucoside	29	869	1.5
62	23.83	177.0552	0.00	C_10_H_10_O_3_	159.0444 (97), 131.0495 (5), 115.0547 (100)	Dihydroxytetralone	158	3117	2.3
63	23.83	337.0930	2.08	C_16_H_18_O_8_	191.0543 (71), 163.0404 (21), 145.0323 (9), 119.0505 (21), 93.0336 (100)	Coumaroyl quinic acid	31	892	1.6
64	24.67	469.1361	3.20	C_21_H_26_O_12_	175.0397 (100)	Trihydroxynaphthalene pentosyl-hexoside	43	766	2.5
65	25.09	469.1342	−0.85	C_21_H_26_O_12_	175.0391 (100)	Trihydroxynaphthalene pentosyl-hexoside			
66	25.64	385.1148 ^8^	3.38	C_16_H_20_O_8_	339.1042 (27), 177.0532 (15), 159.0437 (100)	Dihydroxytetralone hexoside	62	571	5.0
67 ^6^	25.82	337.0916	−2.08	C_16_H_18_O_8_	176.0446 (16), 175.0390 (100)	1,4,8-Trihydroxynaphthalene 1-*O*-β-d-glucoside			
68	25.91	635.0859	−3.94	C_27_H_24_O_18_	465.0638 (69), 313.0571 (67), 169.0141 (100), 125.0223 (12), 124.0127 (18)	Tri-*O*-galloyl-glucose			
69	26.77	193.0501		C_10_H_10_O_4_	175.0382 (100), 157.0277 (30), 147.0479 (11), 131.0458 (18)	Trihydroxytetralone	102	2745	1.7
70	27.32	281.0666	1.78	C_13_H_14_O_7_	163.0408 (62), 135.0289 (14), 119.0494 (100), 117.0329 (26), 75.0079 (9)	Coumaroyl threonic acid	232	3414	3.1
71 ^6^	27.88	163.0400	3.07	C_9_H_8_O_3_	119.0492 (100)	*p*-Coumaric acid	108	582	8.4
72	28.11	511.1453	0.20	C_23_H_28_O_13_	341.0830 (11), 327.0719 (12), 197.0480 (14), 182.0204 (10), 169.0462 (27), 154.0245 (13), 153.0187 (100)	Hydroxy-dimethoxyphenol syringoyl-glucoside	73	1060	3.1
73	28.91	507.1152	2.56	C_23_H_24_O_13_	313.0579 (48), 193.0500 (37), 175.0394 (33), 169.0117 (100), 157.0293 (8), 125.0233 (31)	Trihydroxytetralone galloyl-hexoside			
74	29.29	311.0761	−1.93	C_14_H_16_O_8_	193.0466 (14), 149.0554 (11), 134.0359 (100), 117.0334 (21), 75.0080 (6)	Feruloyl threonic acid			
75	30.05	403.1613	2.23	C_18_H_28_O_10_	223.0982 (22), 179.1072 (100), 161.0975 (16)	Glucopyranose, 1-[10-hydrogen (2E,4E)-8-hydroxy-2,7-dimethyl-2,4-decadienedioate]			
76 ^6^	30.30	300.9989	1.66	C_14_H_6_O_8_	283.9921 (15), 271.9869 (8), 257.0076 (12), 245.0128 (9)	Ellagic acid	220	2775	3.6
77	30.34	511.1074	−2.74	C_22_H_24_O_14_	197.0448 (14), 182.0280 (14), 169.0150 (20), 168.0060 (40), 149.9941 (100), 138.0327 (12), 125.0228 (26), 124.0165 (18)	Syringoyl-galloyl-*O*-glucose			
78	30.38	481.1357	2.29	C_22_H2_6_O_12_	341.0879 (40), 197.0433 (16), 182.0264 (8), 152.0482 (33) , 138.0316 (80), 123.0084 (100)	Hydroxy-methoxyphenol syringoyl-glucopyranoside	42	427	4.5
79	31.10	509.1296	0.20	C_23_H_26_O_13_	341.0851 (14), 327.0708 (30), 197.0458 (46), 167.0349 (100), 152.0126 (44), 137.0244 (28), 123.0442 (15), 108.0197 (26)	Vanilloyl-*O*-syingoyl-glucose	51	680	3.4
80	31.31	539.2120 ^8^	−1.67	C_25_H_34_O_10_	493.2079 (100), 361.1590 (66), 179.0692 (34), 165.0559 (32)	Secoisolariciresinol pentoside			
81	31.89	495.1161	4.44	C_22_H_24_O_13_	451.1234 (8), 327.0654 (13), 183.0281 (19), 169.0136 (8), 152.0119 (100), 138.0324 (56), 108.0210 (100)	Vanilloyl-methylgalloyl-*O*-glucose	93	1211	3.5
82	32.23	433.0783	2.77	C_20_H_18_O_11_	287.0436 (7), 281.0692 (7), 169.0144 (39), 163.0391 (72), 135.0306 (100), 125.0224 (66), 119.0480 (68), 117.0180 (8), 107.0131 (40), 75.0085 (48)	Coumaroyl-galloyl-threonic acid			
83	33.99	361.1660	2.49	C_20_H_26_O_6_	343.1534 (16), 179.0698 (80), 165.0545 (31), 163.0760 (36), 145.0644 (76), 135.0434 (49), 121.0293 (62), 107.0499 (63), 93.0352 (71)	Secoisolariciresinol	182	2705	3.1
84	34.06	491.1183	−1.43	C_23_H_24_O_12_	313.0604 (9), 211.0225 (38), 177.0617 (10), 169.0158 (94), 159.0436 (61), 125.0225 (69), 124.0159 (100)	Dihydroxytetralone galloy-hexoside			
85 ^6^	34.37	447.0920	−1.57	C_21_H_20_O_11_	301.0338 (100), 151.0017 (71)	Quercetin-3-*O*-α-l-rhamnoside			
86 ^7^	34.96	391.1757	0.00	C_21_H_28_O_7_	193.0861 (43), 175.0776 (57), 161.0558 (76), 135.0457 (100), 123.0461 (66)	Juglanol B	94	886	4.8
87 ^7^	35.61	619.1292	−1.13	C_28_H_28_O_16_	325.0337 (85), 324.0248 (100)	2,3,7,11,12-pentahydroxy-6-oxabenzo[*α*]anthracen-5-one pentosyl-hexoside			
88	37.77	535.1457	0.93	C_25_H_28_O_13_	341.0897 (35), 197.0454 (20), 193.0490 (100), 175.0405 (64), 137.0231 (13), 125.0212 (8)	Trihydroxytetralone syringoyl-hexoside	88	1230	3.3
89 ^7^	39.96	551.2145 ^8^	2.90	C_26_H_34_O_10_^−^	505.1992 (18), 343.1520 (28), 325.1420 (79), 307.1235 (9), 89.0246 (100)	Jugcathayenoside	28	249	5.1
90 ^7^	41.05	549.1990 ^8^	3.28	C_26_H_32_O_10_^−^	503.1977 (6), 341.1364 (18), 323.1282 (100), 295.1367 (9)	Juglaside A	56	865	2.9
91 ^7^	41.47	311.0193	0.32	C_16_H_8_O_7_	267.0289 (52), 223.0364 (34), 195.0462 (100), 167.0454 (27)	Hydroxyanthraquinone dicarboxylic acid			
92 ^7^	43.95	343.1550	1.46	C_20_H_24_O_5_	179.0705 (100), 121.0297 (49)	Anhydrosecoisolariciresinol (or its isomer)			
93 ^7^	45.34	267.0302	3.37	C_15_H_8_O_5_	223.0402 (33), 195.0440 (100)	Hydroxyanthraquinone carboxylic acid			

^1^ Data were acquired over a range of *m*/*z* 100–2000 for MS; ^2^ Data were acquired over a range of *m*/*z* 50–2000 for MS/MS; ^3^ Those compounds with stereo centers which configuration can’t be determined only by MS data was tentatively identified as the one that universally exist as natural products; ^4^ The absorption ration was calculated using the following formula: $\mathrm{Absorption}\;\mathrm{ratio}\;(\%)=\frac{\mathrm{Peak}\;\mathrm{area}\;\mathrm{in}\;\mathrm{medicated}\;\mathrm{egg}\;\mathrm{solution}\times 0.1}{\mathrm{Peak}\;\mathrm{area}\;\mathrm{in}\;\mathrm{decoction}\times 0.22}\times 100\%$; ^5^ HHDP: hexahydroxy-diphenoyl; ^6^ Peaks that were undoubtedly identified by comparing with reference compounds; ^7^ Peaks that were tentatively identified due to the lack of reference compounds and mass data in literature; ^8^ Peaks that formed formiate adduct ions of [M + HCOO]^−^.

## Supplementary Materials

The following are available online, Figure S1: Representation of the fragmentation pattern of (a) tannins (Peak 41), (b) naphthalene derivatives (Peak 62) and (c) organic acids (Peak 7), Figure S2: Proposed fragmentation pattern of the compounds tentatively identified: (a) Peak 38, (b) Peak 86, (c) Peak 87, (d) Peak 89, (e) Peak 90, (f) Peak 91 and (g) Peak 92, Figure S3: (a) EIC of the *Juglans mandshurica* decoction and the blank and medicated egg solutions at *m*/*z* 203 in MS and (b) CID MS/MS spectra (obtained at an energy of −20 eV) of the ion at *m*/*z* 203 in the *Juglans mandshurica* decoction and the medicated egg solution, Figure S4: EIC of the *Juglans mandshurica* decoction and the blank and medicated egg solutions at *m*/*z* 483 in MS, Table S1: Information for the reference compounds.
