# Supplementary material for: Identification of Polar Constituents in the Decoction of Juglans mandshurica and in the Medicated Egg Prepared with the Decoction by HPLC-Q-TOF MS2
Source: Molecules. 2017 Sep 1;22(9):1452. doi: 10.3390/molecules22091452 (PMC6151821; doi:10.3390/molecules22091452)
Supplement: Supplementary file 1 [file molecules-22-01452-s001.pdf]

molecules

MDPI

Supplementary Materials

Identification of Polar Constituents in the Decoction of *Juglans mandshurica* and in the Medicated Egg Decocted with the Decoction by HPLC-Q-TOF MS²

Tian-Min Wang [1], Ying Fu [1], Wen-Jie Yu [1], Chen Chen [1], Xue Di [1], Hui Zhang [1], Yan-Jun Zhai [1,*], Zheng-Yun Chu [1], Ting-Guo Kang [1] and Hu-Biao Chen [2,*]

[1] School of Pharmacy, Liaoning University of Traditional Chinese Medicine, Dalian 116600, China; wang_tm@163.com (T.-M.W); Ying.Fu@quintiles.com (Y.F.); crystal_5yu@163.com (W.-J.Y.); MOC-CHEN@163.com (C.C.); dixhappy@163.com (X.D.); syyycs@163.com (H.Z.); chuzhengyun@163.com (Z.-Y.C.); kangtg@lnutcm.edu.cn (T.-G.K.)

[2] School of Chinese Medicine, Hong Kong Baptist University, Kowloon Tong 999077, Hong Kong, China;

* Correspondence: lnzyzyj@sohu.com (Y.-J.Z.); hbchen@hkbu.edu.hk (H.-B.C.); Tel.: +86-411-85890138 (Y.-J.Z.); +852-34112060 (H.-B.C.)

Academic Editor: name
Received: date; Accepted: date; Published: date

Table S1 Information for reference compounds.

No.	Reference compound	Structural type	Concentration (μg/mL)	Standard solution	t_R (min)
1	Gallic acid	Organic acid	21	A	7.9
2	Protocatechuic acid	Organic acid	36	A	13.1
3	Chlorogenic acid	Organic acid	22	A	19.5
4	Caffeic acid	Organic acid	15	B	21.8
5	Syringic acid	Organic acid	15	A	22.6
6	1,2,6-Tri-O-galloyl-*β*-D-glucose	Tannin	35	A	22.7
7	1,4,8-Trihydroxy-naphthalene-1-O-*β*-D -glucoside	Naphthalene	35	A	24.4
8	*p*-Coumaric acid	Organic acid	25	B	27.5
9	1,2,3,6-Tetra-O-galloyl-*β*-D-glucose	Tannin	35	A	29.2
10	Rutin	Flavonoid	106	B	29.6
11	Ellagic acid	Organic acid	10	B	30.0
12	Sinapic acid	Organic acid	20	B	30.3
13	Quercetin-3-O-*α*-L-rhamnoside	Flavonoid	56	A	34.2
14	Kaempferol-3-O-*α*-L-rhamnoside	Flavonoid	56	A	37.9
15	Luteolin	Flavonoid	116	B	42.4
16	Quercetin	Flavonoid	70	A	42.6
17	Juglone	Naphthoquinone	45	A	43.9
18	Apigenin	Flavonoid	62	B/A	45.6
19	Kaempferol	Flavonoid	98	A/B	46.2
20	Acacetin	Flavonoid	110	A	52.8

(a)

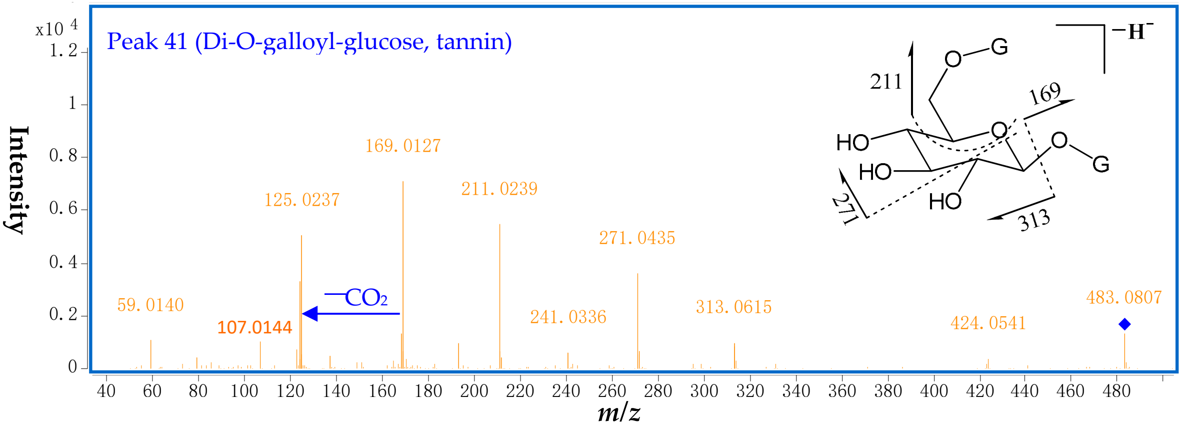

(b)

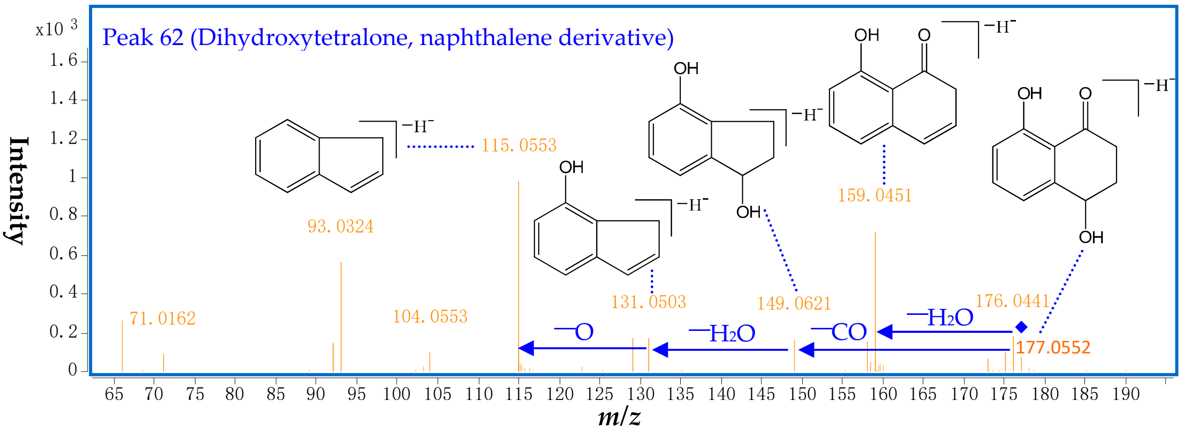

(c)

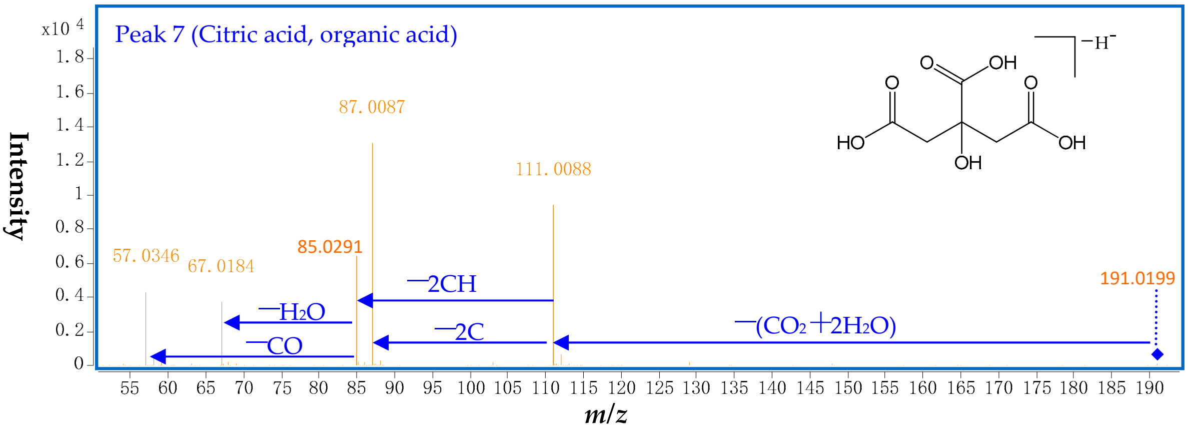

Figure S1 Representation of the fragmentation pattern of (**a**) tannins (Peak 41), (**b**) naphthalene derivatives (Peak 62) and (**c**) organic acids (Peak 7).

(a)

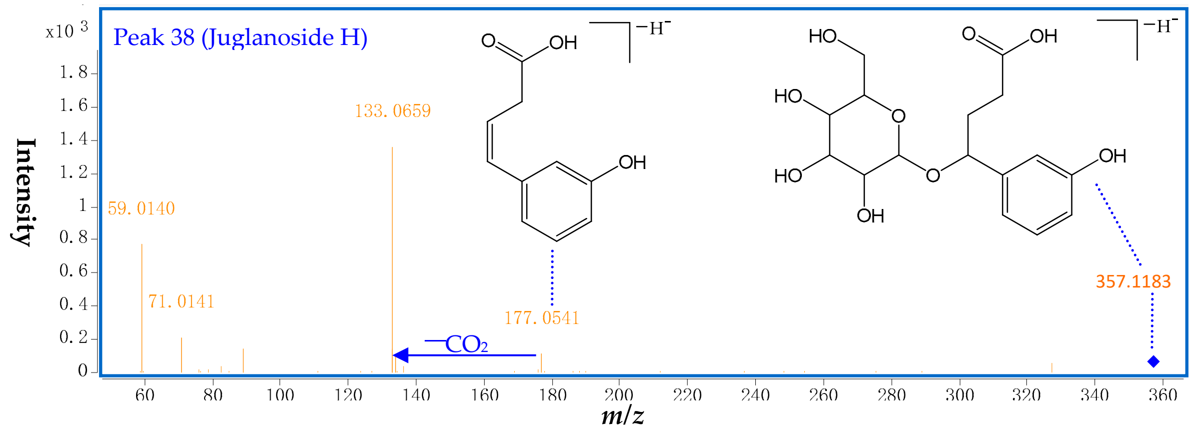

Peak 38 (Juglanoside H)
x10 3
Intensity
133. 0659
59. 0140
71. 0141
177. 0541
357.1183
—CO2
m/z

(b)

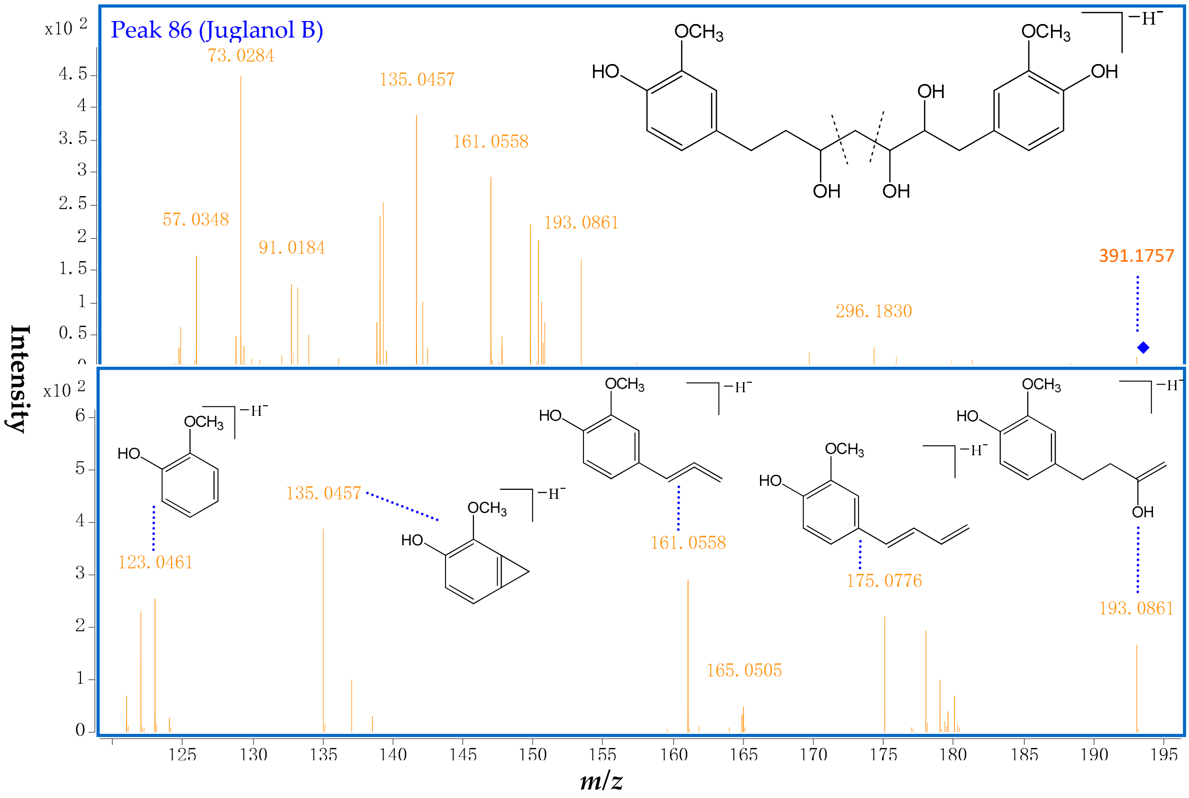

Peak 86 (Juglanol B)
x10 2
Intensity
73. 0284
135. 0457
161. 0558
57. 0348
91. 0184
193. 0861
391.1757
296. 1830
123. 0461
175. 0776
165. 0505
m/z

(c)

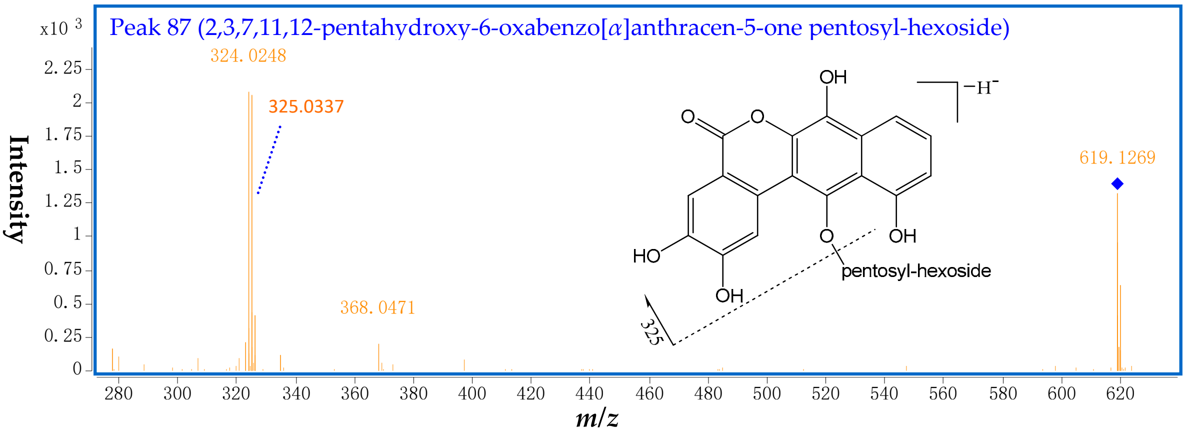
Peak 87 (2,3,7,11,12-pentahydroxy-6-oxabenzo[α]anthracen-5-one pentosyl-hexoside)
x10 3
Intensity
324. 0248
325.0337
368. 0471
619. 1269
pentosyl-hexoside
325
m/z

(d)

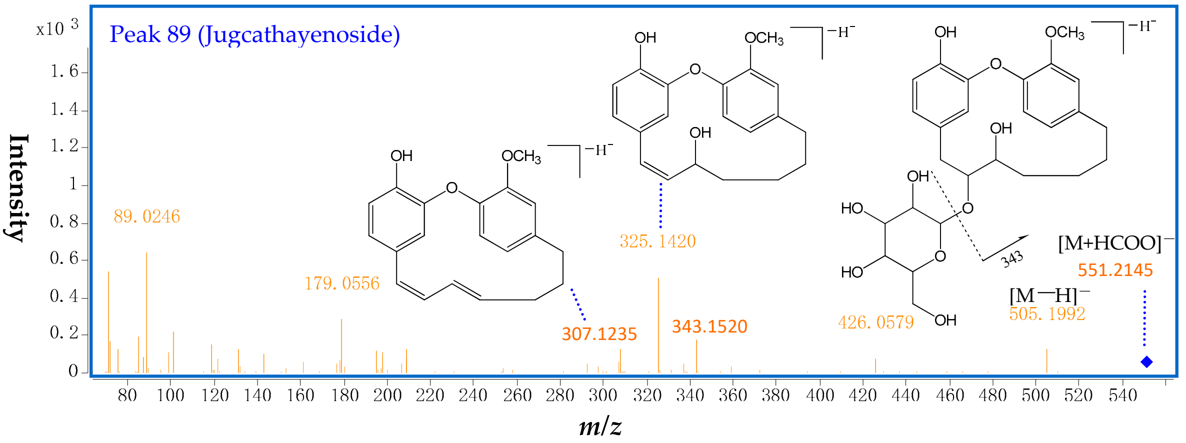
Peak 89 (Jugcathayenoside)
x10 3
Intensity
89. 0246
179. 0556
307.1235
325. 1420
343.1520
426. 0579
[M+HCOO]⁻
551.2145
[M−H]⁻
505. 1992
343
m/z

(e)

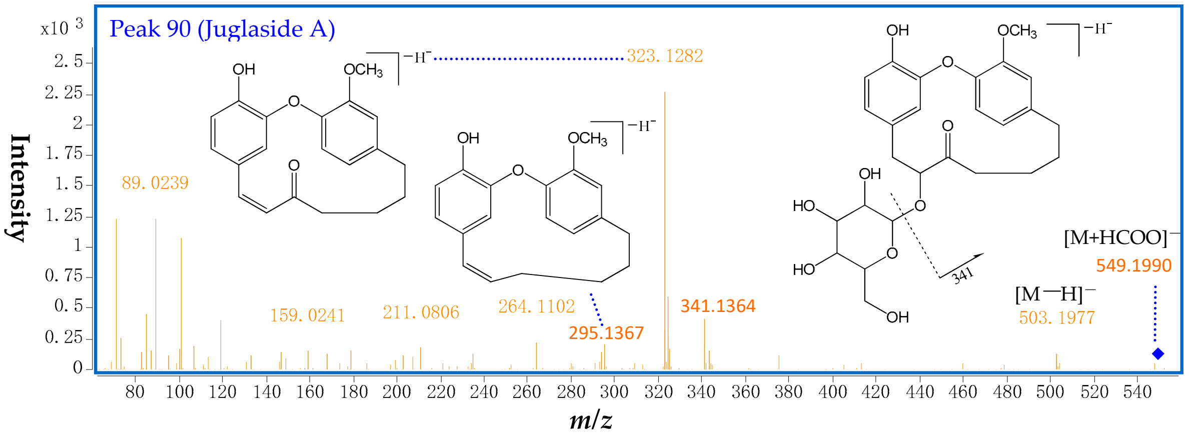
Peak 90 (Juglaside A)
x10 3
Intensity
323. 1282
89. 0239
159. 0241
211. 0806
264. 1102
295.1367
341.1364
[M+HCOO]⁻
549.1990
[M−H]⁻
503. 1977
341
m/z

(f)

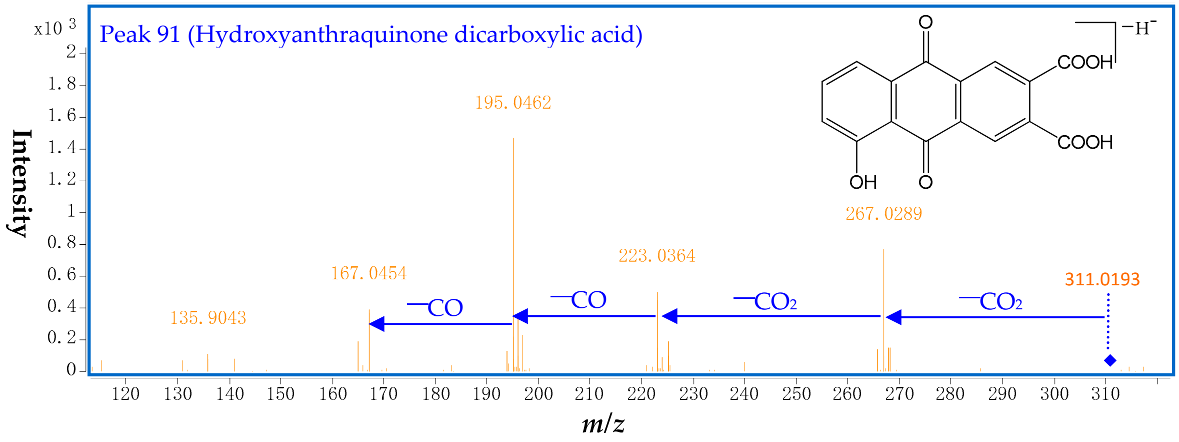

(g)

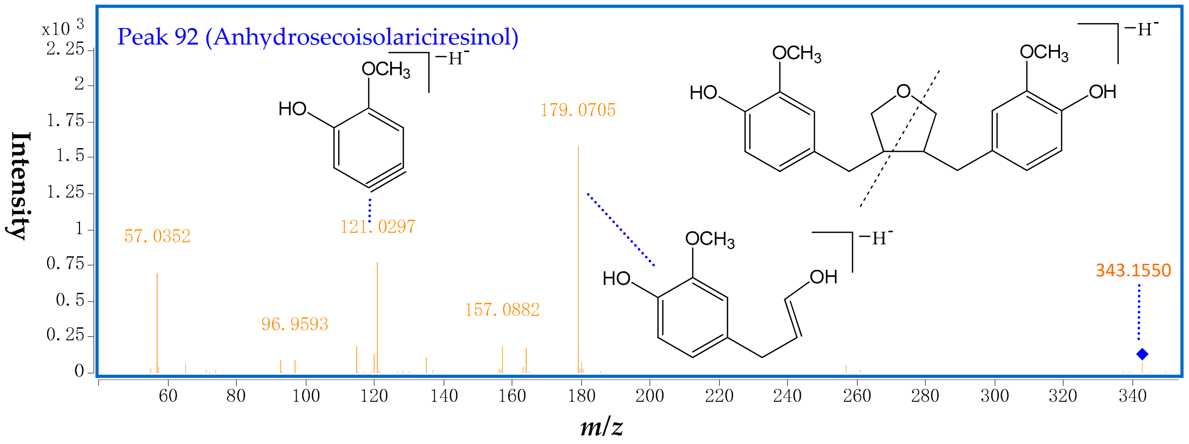

Figure S2 Proposed fragmentation pattern of the compounds tentatively identified: (**a**) Peak 38, (**b**) Peak 86, (**c**) Peak 87, (**d**) Peak 89, (**e**) Peak 90, (**f**) Peak 91 and (**g**) Peak 92.

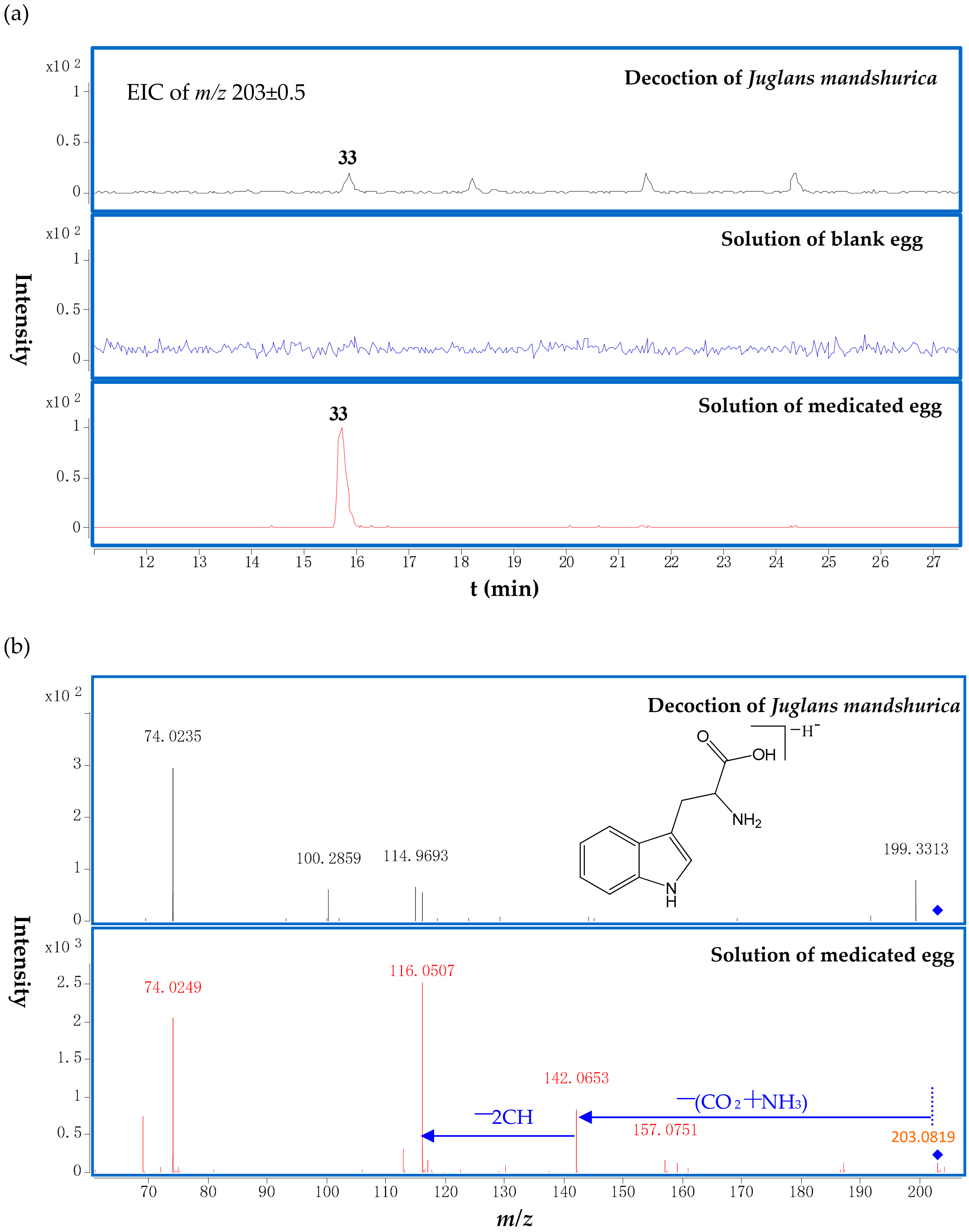

Figure S3 (**a**) EIC of the *Juglans mandshurica* decoction and blank and medicated egg solutions at *m/z* 203 in MS and (**b**) CID MS/MS spectra (obtained at an energy of −20 eV) of the ion at *m/z* 203±0.5 in the *Juglans mandshurica* decoction and medicated egg solution. Peak 33 was identified as tryptophan.

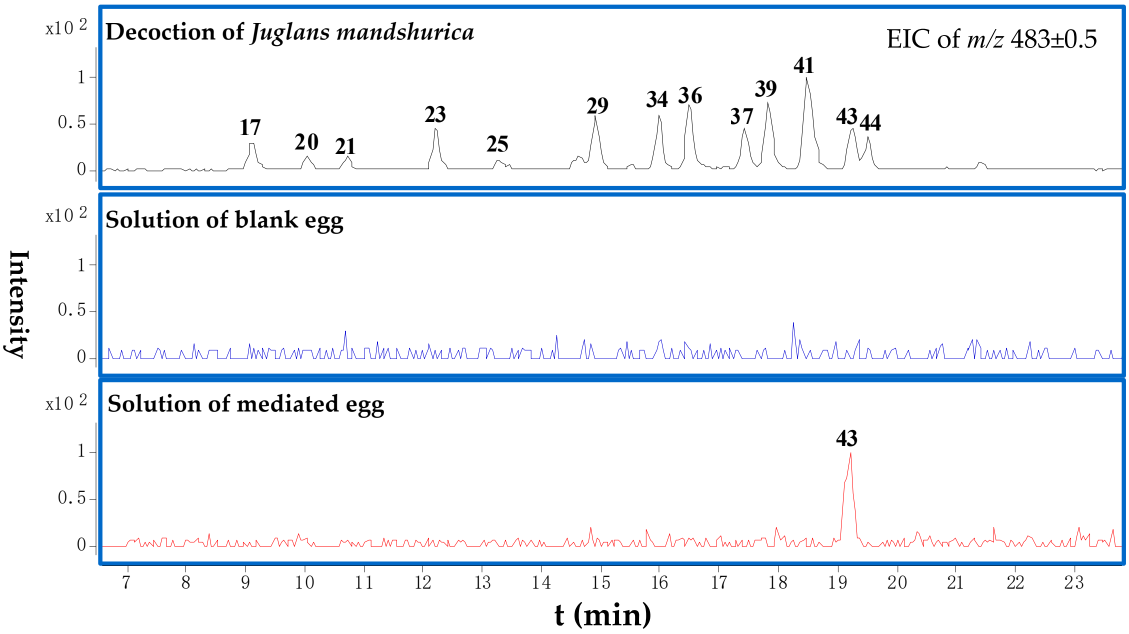

Figure S4 EIC of the *Juglans mandshurica* decoction and blank and medicated egg solutions at *m/z* 483±0.5 in MS. Peaks 43 was identified as hydroxy-dimethoxyphenol galloyl-glucoside, and other peaks were identified as isomers of di-O-galloyl-glucose.

© 2017 by the authors. Submitted for possible open access publication under the terms and conditions of the Creative Commons Attribution (CC BY) license (http://creativecommons.org/licenses/by/4.0/).
